# Effective Induction Programme for Higher Specialist Trainees: A Quality Improvement Project

**DOI:** 10.1192/bjo.2024.404

**Published:** 2024-08-01

**Authors:** Zaib un Nisa, Deepa Krishnan, Kehinde Junaid, Lori Edwards Suárez, Sudheer Lankappa

**Affiliations:** Nottinghamshire Healthcare NHS Foundation Trust, Nottingham, United Kingdom

## Abstract

**Aims:**

To create a safe and effective induction programme for Higher Specialist Trainees (HST) at Nottinghamshire Healthcare NHS Foundation Trust.

An effective induction improves trainees' satisfaction, they feel welcomed and valued. It improves patient safety, retention, and recruitment (GMC Report 2020).

**Methods:**

Based on GMC report, published in 2020, a survey was developed locally and data for 2021 HST induction was collected using digital platform. Initial stakeholder analysis completed, and relevant parties were invited to share the results. Two key deliverables were identified after consultation, one was a dedicated induction programme for HST which was co-produced along with trainees and stakeholders. The other deliverable was updating the induction booklet. The proposed induction plan was implemented in August 2023, the survey was repeated to the new HST cohort following induction via digital platform. Results of the survey were analysed via mixed methods (qualitative & quantitative).

**Results:**

The surveys conducted in 2021 and 2023 were compared and there was an increase in response rate from 50% to 64%. The domains were devised from GMC standards and assessed by if staff had received everything in the domain within a week of starting their placement and results evaluated using a t-test.

Domain A is gaining access to places and system (keys, fobs, security passes, computers, ID badges, mobile phones, IT system). This significantly improved from 27% to 88% with a p-value of < 0.001.

Domain B is physical orientation of the setting (staff facilities such as lockers, parking, library, and site layout). This significantly improved from 45% to 88% with a p-value of < 0.018.

Domain C is gaining day to day knowledge (HR, rota, annual leave, study leave, pay-roll, mandatory training, e-expenses, and guardian of safe working). There was no significant change between 9% and 19% with a p-value of < 0.48.

Domain D is an understanding of expectations (duties and responsibility during working hours, on-call, team introduction). This significantly improved from 9% to 69% with a p-value of < 0.002.

HSTs were given the chance to add comments and the responses in 2023 were more positive “excellent induction compared to previous years” compared with 2021 when HSTs felt isolated and devalued “worst ever induction in whole career in NHS”.

**Conclusion:**

Overall, the results of the 2023 survey showed considerable improvement in all the key areas of induction within one week of starting the placement. Domain C demonstrates a challenge still and needs further work.

